# A case of leukocytoclastic vasculitis in a patient on dupilumab

**DOI:** 10.1016/j.jdcr.2023.08.048

**Published:** 2023-09-24

**Authors:** Michelle S. Bach, Brett H. Keeling, Elizabeth Yim

**Affiliations:** a University of Texas at Austin, Dell Medical School, Austin, Texas; b Division of Dermatology, University of Texas at Austin, Dell Medical School, Austin, Texas

**Keywords:** adverse reaction, biologic treatment for atopic dermatitis, dupilumab, leukocytoclastic vasculitis

## Introduction

Dupilumab is a fully human monoclonal IgG4 antibody that inhibits the signaling of IL-4 and IL-13 by targeting the IL-4 alpha receptor.^1^ The most common adverse effect is dupilumab-associated ocular surface disease (DAOSD); however, prior reports of dermatologic complications include erythema nodosum, erythema of the neck and face, and pruritus and peeling of the skin.^2^, ^3^, ^4^ We present a patient with a history of Graves’ disease, fibromyalgia, migraines, subacute cutaneous lupus erythematosus in remission, and atopic dermatitis, who developed leukocytoclastic vasculitis 10 days after starting dupilumab.

## Case report

A 58-year-old woman with atopic dermatitis (AD) presented with a 6-day history of a pruritic and painful rash on the bilateral lower extremities and trunk 10 days after receiving her first loading dose of dupilumab. The rash first emerged on the neck and abdomen and then appeared on the lower extremities. The patient completed a second dose of dupilumab during the course of her rash. On review of systems, the patient denied fevers, chills, new illnesses, or shortness of breath; however, she reported a worsening headache with associated photophobia. She has a history of migraines and a cerebral aneurysm several years prior requiring clipping. Her migraines were under adequate control until the onset of her rash. The patient was referred to her neurologist for further evaluation and imaging which returned unremarkable.

Past medical history was significant for Graves’ disease, fibromyalgia, migraines, and subacute cutaneous lupus erythematosus (SCLE). Past serologies were weakly positive for anti-ku and positive for anti-nuclear antibody (ANA) of 1:160. The patient was not on any treatments for her lupus, which was in remission. She also has a history of atopic dermatitis, supported by biopsies that showed spongiotic dermatitis in the past. Prior treatment for her AD included topical steroids, topical tacrolimus, methotrexate, as well as dupilumab, which she had been on for at least a year, before self-discontinuing given improvement of her AD.

**Fig 1:**
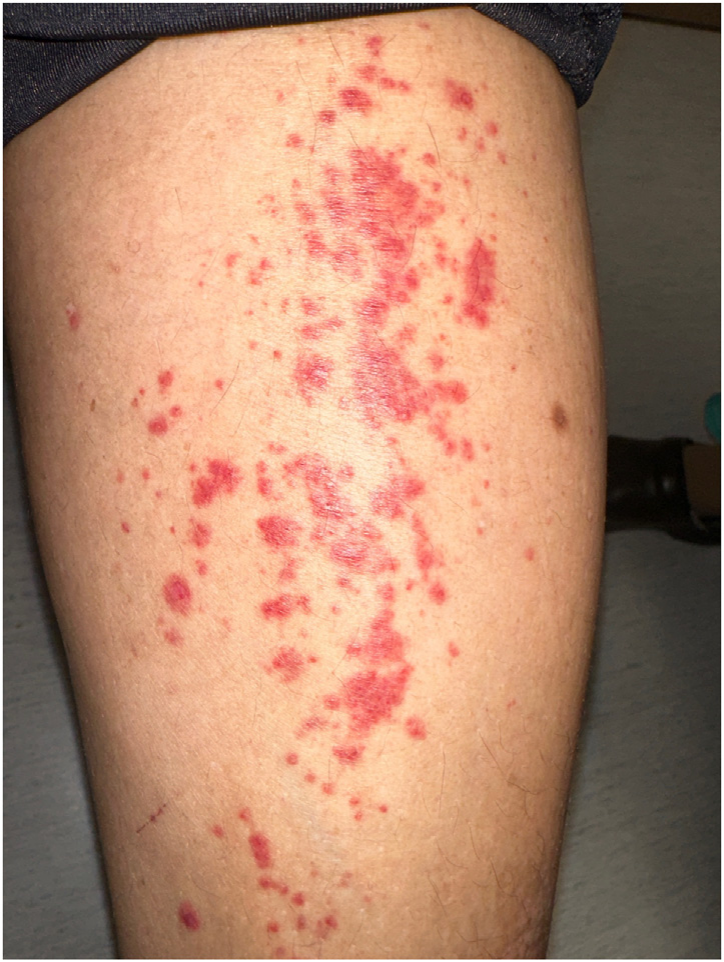
Clinical presentation. Palpable nonblanching purpura of the lower extremities.

**Fig 2:**
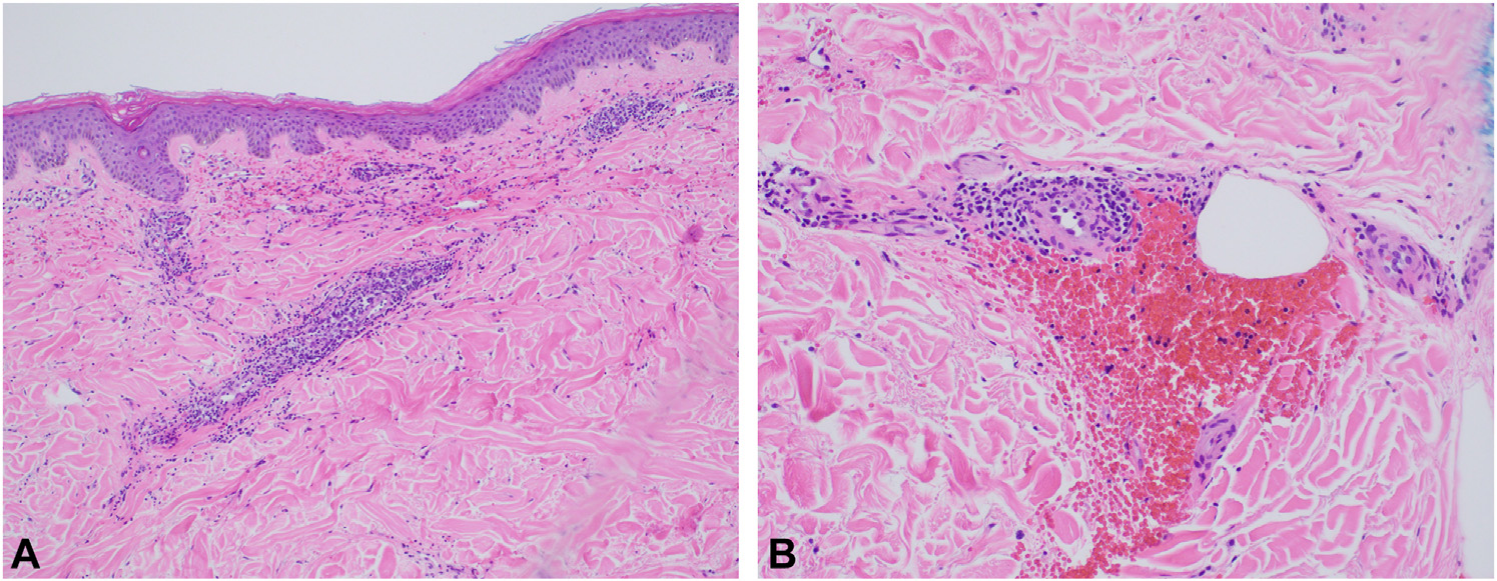
Histopathological examination of the left lower extremity demonstrates abundant extravasation of erythrocytes in surrounding vessels within the dermis, extensive infiltration of vessel walls by mixed inflammation, including lymphocytes, histiocytes, neutrophils, and multiple eosinophils, and nuclear dust consistent with karyorrhexis (**A** and **B**). Minimal fibrinoid change was seen within occasional inflamed superficial dermal vessel walls. All features were consistent with leukocytoclastic vasculitis (Hematoxylin-eosin stain; 10×, and 20× respectively).

The patient presented to our clinic a year later with worsening AD supported on repeat biopsy. Given her history of improvement on dupilumab, the patient was restarted on dupilumab and then 10 days later developed new-onset palpable purpura involving the bilateral lower extremities (Fig 1). A punch biopsy of the left anterior leg was performed, and histopathology showed abundant extravasation of erythrocytes in surrounding vessels within the dermis with karyorrhexis consistent with leukocytoclastic vasculitis (Fig 2). An extensive work-up was performed that revealed a repeat ANA of 1:80, mildly elevated complements, negative rheumatoid factor, negative anti-streptolysin O titer, negative hepatitis panel, negative urinalysis, and a complete blood count (CBC) and comprehensive metabolic panel (CMP) that were within normal limits. Her serum electrophoresis was negative. The patient was initially offered prednisone; however, after meeting with her neurologist about her migraines, she was switched to a course of methylprednisolone with taper. Her dupilumab was discontinued. At her 2 week follow up, her palpable purpura had completely resolved.

## Discussion

Dupilumab is approved by the Food and Drug Administration (FDA) for the treatment of moderate to severe atopic dermatitis in patients ≥6 m of age who are unresponsive to topical medications.^5^^,^^6^ The most common side effect of dupilumab in AD patients is dupilumab-associated ocular surface disease (DAOSD).^6^ Less commonly reported adverse events include headaches, nasopharyngitis, hypersensitivity reactions, arthralgias, and eosinophilia. Cutaneous adverse events of dupilumab include erythema nodosum, on-and-off neck and facial rash, pruritus and peeling of the skin, eczema, alopecia areata, and cutaneous T cell lymphoma.^2^, ^3^, ^4^^,^^7^ Although other biologics including infliximab, adalimumab, secukinumab, rituximab, ustekinumab, and tocilizumab have been associated with leukocytoclastic vasculitis (LCV), cases of dupilumab-associated LCV have not yet been reported in the literature.^8^

Leukocytoclastic vasculitis, also known as cutaneous small vessel vasculitis, is the most common form of cutaneous vasculitis that involves deposition of immune complexes in blood vessels, largely in the postcapillary venule, and recruitment of neutrophils causing leukocytoclasia and destruction of the involved vasculature.^9^ LCV typically presents clinically as palpable purpura, but can also present with urticarial, vesicular, or targetoid lesions. Confirmatory diagnosis is via histopathology. Most cases of LCV are idiopathic but can also be associated with drugs, infections, collagen-vascular diseases, hematologic disorders, or malignancy.^9^^,^^10^ Patients with suspected LCV require a comprehensive work-up including CBC, CMP, hepatitis panel, complements, autoantibodies, and urinalysis to rule out systemic disease, which is seen in ∼50% of patients. Treatment of LCV is determined if it is skin-limited or systemic, but most idiopathic cases of cutaneous LCV resolve with rest, elevation, and NSAIDS with systemic corticosteroids reserved for refractory or complicated cases.^10^ If a drug is the cause for LCV, withdrawal of the drug is important.

The pathogenesis of LCV in our patient receiving dupilumab is unclear; however, suppression of IL-4 and IL-13 may have led to a neutrophilic inflammatory response specifically targeting the vasculature. While we considered our patient’s history of SCLE as a possible trigger for LCV, this was ruled out given our patient’s repeat ANA of 1:80, and she also exhibited hypercomplementemia of C3 and C4, when we would expect to see hypocomplementemia in relation to SCLE. Given the temporal relationship between the start of her dupilumab and emergence of LCV 10 days after the first loading dose as well as resolution of her symptoms after discontinuing the biologic, we propose a potential association between dupilumab and LCV. In conclusion, this case highlights the need for dermatologists to be made aware that LCV could occur in the setting of prescribing this medication to patients.

## Conflicts of interest

None disclosed.

## References

[1] Thibodeaux Q., Smith M.P., Ly K., Beck K., Liao W., Bhutani T. (2019). A review of dupilumab in the treatment of atopic diseases. Hum Vacc Immunother.

[2] Mustin D.E., Cole E.F., Blalock T.W., Kuruvilla M.E., Stoff B.K., Feldman R.J. (2022). Dupilumab-induced erythema nodosum. Jaad Case Rep.

[3] Albader S.S., Alharbi A.A., Alenezi R.F., Alsaif F.M. (2019). Dupilumab side effect in a patient with atopic dermatitis: a case report study. Biologics Targets Ther.

[4] Hammadi A.A., Parmar N.V. (2019). Erythema, pruritus, and diffuse peeling of skin during dupilumab therapy for atopic dermatitis in three adults. Int J Dermatol.

[5] Grinich E.E., Simpson E.L. (2020). Comprehensive Dermatologic Drug Therapy.

[6] Dupixent FDA package insert. https://www.accessdata.fda.gov/drugsatfda_docs/label/2022/761055s044lbl.pdf.

[7] Bettuzzi T., Drucker A., Staumont-Sallé D., Bihan K., Lebrun-Vignes B., Sbidian E. (2022). Adverse events associated with dupilumab in the World Health Organization pharmacovigilance database. J Am Acad Dermatol.

[8] da Silva Cendon Duran C., da Paz A.S., Santiago M.B. (2021). Vasculitis induced by biological agents used in rheumatology practice: a systematic review. Arch Rheumatol.

[9] Tomasini C.F., High W.A., Argenziano G., Zalaudek I. (2018). Dermatology.

[10] Fraticelli P., Benfaremo D., Gabrielli A. (2021). Diagnosis and management of leukocytoclastic vasculitis. Intern Emerg Med.

